# Vaping Among Malaysian Students 2022–2024: A Systematic Review of Prevalence, Awareness and Influencing Factors

**DOI:** 10.21315/mjms-04-2025-261

**Published:** 2025-10-31

**Authors:** Vitalis Ronald Eden, Hazrina Hamid, Mohd Sabri Mohamad, Sreemoy Kanti Das, Regidor Iii Dioso

**Affiliations:** 1 Faculty of Pharmacy, Lincoln University College, Petaling Jaya, Selangor, Malaysia; 2 Department of Pharmacy, Faculty of Allied Health Sciences, IJN University College, Kuala Lumpur, Malaysia; 3 Department of Medical and Health Sciences, Faculty of Allied Health Sciences, IJN University College, Kuala Lumpur, Malaysia; 4 Faculty of Nursing, Lincoln University College, Petaling Jaya, Selangor, Malaysia

**Keywords:** systematic review, e-cigarettes, vaping, prevalence, Malaysian students

## Abstract

The increasing prevalence of e-cigarette use among students in Malaysia has raised significant public health concerns. Although various individual studies have explored this issue across different educational settings, a consolidated understanding of usage patterns, awareness, and influencing factors remains limited. This systematic review aims to synthesise the current evidence on the prevalence, knowledge, attitudes, and determinants of e-cigarette use among students in Malaysian higher education institutions. We conducted a systematic search across major databases, including PubMed, Scopus, and Google Scholar, to identify relevant cross-sectional studies published between January 2022 and December 2024. The inclusion criteria focused on peer-reviewed articles involving students from universities and colleges in Malaysia aged 17 to 40 years. Data extraction captured prevalence rates, participant demographics, knowledge and attitudes toward vaping, and key influencing factors. Nine studies, encompassing data from 2,828 students across diverse geographic and academic settings in Malaysia, met the inclusion criteria. The reported prevalence of e-cigarette use ranged from 2.9% to 32%, with considerable variation across regions and academic disciplines. Peer influence emerged as the most frequently cited determinant of use, followed by social trends, media exposure, and stress relief. While general awareness of e-cigarettes and their risks was relatively high, gaps in knowledge and preventive behaviours were consistently observed. Institutions with a healthcare-focused curriculum demonstrated lower prevalence rates and higher knowledge scores. Although awareness of e-cigarette risks is widespread among students, inconsistent knowledge and insufficient behavioural interventions persist, highlighting the need for targeted health education initiatives and peer-led prevention strategies.

## Introduction

The global landscape of tobacco consumption has undergone a significant transformation with the advent of electronic cigarettes, also known as e-cigarettes, which are often perceived as a safer alternative to traditional smoking (1). Technological advancements, marketing strategies, and changing consumer perceptions have led to the evolution of e-cigarettes into a complex and rapidly expanding industry (2). The World Health Organization has raised concerns over the increasing use of e-cigarettes, especially among youth, citing the potential risks of nicotine addiction, dual use with conventional cigarettes, and the unknown long-term health consequences of inhaling aerosolised chemicals (3). In Malaysia, the surge in e-cigarette use has mirrored global trends, particularly among adolescents and young adults in tertiary education. Malaysia ranks among Southeast Asian countries with the growing popularity of vaping products, facilitated by the easy availability of devices, attractive flavourings, and social media influence (4).

Despite existing tobacco control policies under the Control of Tobacco Product Regulations (CTPR), e-cigarettes have not been regulated with equivalent stringency, creating a regulatory grey area that may contribute to their rising appeal among youth (5). Malaysia’s student population, which encompasses public and private institutions, represents a critical demographic for assessing the behavioural and social determinants of e-cigarette use. These young adults are in a transitional life stage characterised by increased autonomy, peer influence, academic stress, and exposure to digital environments, all of which can shape health-related behaviours, including substance use (6). Peer influence, social media promotion, and targeted advertising have shaped Malaysia’s e-cigarette landscape, prompting the enactment of the Control of Smoking Products for Public Health Act 2024 (Act 852), the country’s first comprehensive law regulating tobacco and vaping products. The Act mandates product registration, restricts advertising and sponsorship, enforces labelling requirements, and imposes age-of-sale restrictions and phases in nicotine and pod volume limits, although enforcement gaps, public misperceptions, and illicit market access remain key challenges (7).

Moreover, evidence suggests that e-cigarettes may serve as a gateway to combustible tobacco use and other addictive behaviours, undermining decades of tobacco control efforts (8, 9); this concern is especially pertinent in low- and middle-income countries, where regulatory frameworks for emerging tobacco products are either underdeveloped or inconsistently enforced (10). In Malaysia, the use of e-cigarettes has mirrored global trends, with a marked rise among adolescents and young adults over the past decade. As per the Global Adult Tobacco Survey Malaysia 2011 and the National Health and Morbidity Survey (NHMS) 2019, the prevalence of e-cigarette use has increased significantly among males aged 15 to 24 years (11, 12). Malaysia has become one of the leading Southeast Asian countries with respect to the popularity of vaping products, facilitated by the easy accessibility of devices, appealing flavour profiles, and aggressive digital marketing strategies (13).

E-cigarettes are readily available through online platforms, specialty vape shops, and convenience stores, often with minimal regulatory oversight (14). Despite the presence of tobacco control legislation, such as the CTPR under the Food Act 1983, e-cigarettes fall into a regulatory grey area. Until recently, these devices were not regulated with the same rigour as combustible tobacco products, creating a loophole that may have contributed to their attractiveness, particularly among the youth (15). The absence of consistent enforcement and clear legal status of e-cigarettes in Malaysia has complicated efforts to prevent access for underage consumers and regulate marketing practices (16).

The student population in Malaysia, comprising individuals enrolled in tertiary education institutions including public universities, private colleges, and vocational training centres, represents a key demographic for understanding the social and behavioural determinants of e-cigarette use. Students in this age group are navigating a critical transitional life stage marked by increased independence, identity formation, peer pressure, academic stress, and digital engagement, all of which have been linked to risk-taking behaviours, including substance use (17, 18). The educational setting, with its diverse social environments and health education levels, also plays an important role in shaping students’ vaping perceptions and practices (19). Higher prevalence was reported in male-dominated institutions and among non-healthcare students. Factors consistently associated with e-cigarette use in these studies include peer influence, curiosity, perceived stress relief, internet exposure, and social media trends (20).

Furthermore, awareness and knowledge of the health risks associated with vaping are often limited, especially among non-medical students (21–24). In contrast, studies involving students from medical, nursing, or dental faculties have shown higher awareness levels and more cautious attitudes toward e-cigarette use, although experiments have still been conducted (25). Even among these populations, the perception of vaping as a less harmful alternative to smoking remains prevalent, reflecting gaps in health education or misinformation propagated through informal channels (26). Most studies use self-administered questionnaires and are limited to specific institutions or geographic locations, thereby restricting generalizability and national-level insights. Therefore, this systematic review aimed to provide a comprehensive understanding of the vaping landscape in Malaysian higher education by consolidating recent evidence on the prevalence, knowledge, attitudes, and influencing factors of e-cigarette use among students from multiple institutions across the country.

## Methods

### Study Design

This systematic review was conducted to assess the prevalence, awareness, knowledge, attitudes, and contributing factors of e-cigarette use among Malaysian tertiary education students. The review adhered to the Preferred Reporting Items for Systematic Reviews and Meta-Analyses (PRISMA) 2020 statement, which provides updated guidance on eligibility criteria, evidence appraisal, and flow diagram reporting to enhance methodological transparency and rigour (26). The PRISMA framework was selected for its applicability to quantitative and mixed-method public health studies, making it particularly suitable for synthesising cross-sectional data and summarising patterns in prevalence, behaviours, and risk factors. This structured approach ensures reproducibility, comparability, and comprehensive reporting across similar reviews.

The search strategy was independently validated by a second reviewer to ensure completeness and accuracy. Reference lists of relevant articles were also hand-searched to identify additional studies. Two reviewers independently evaluated the methodological quality, with disagreements resolved by consensus. Findings from studies with a high risk of bias were not excluded but were interpreted with caution, and their limitations were explicitly considered when synthesising evidence. Peer-reviewed journal articles and grey literature, including government reports, theses, and conference abstracts, were screened to minimise publication bias.

### Eligibility Criteria

Inclusion criteria encompassed original cross-sectional studies conducted in Malaysia and published between January 2022 and December 2024. Eligible studies focused on university, college, or vocational students and reported data on at least one of the following variables: prevalence of e-cigarette use, awareness, knowledge, attitudes, or associated factors. Only studies published in English and available as full texts were considered. Exclusion criteria comprised studies involving nonstudent populations, those addressing only traditional cigarette use without reference to e-cigarettes, qualitative or interventional designs, editorials, letters, reviews, and conference abstracts lacking full methodological and results sections.

### Information Sources and Search Strategy

A systematic literature search was conducted in four major electronic databases: PubMed, Scopus, Web of Science, and Google Scholar. Supplementary manual searches of institutional repositories and grey literature were also performed. The search period was from January 2024 to February 2024. Keywords and MeSH terms were applied in various combinations: (“e-cigarette” OR “vaping” OR “vape” OR “electronic nicotine delivery systems”) AND (“students” OR “university” OR “college” OR “tertiary education”) AND (“Malaysia”). Boolean operators were used to optimise sensitivity and specificity, and filters were applied to retrieve English-language publications within a defined period.

### Study Selection

**Figure 1:**
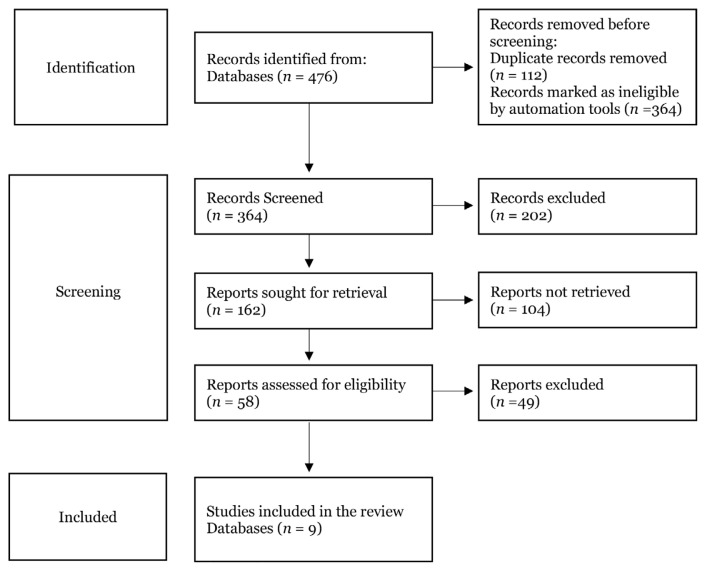
PRISMA flowchart of systematic review

All retrieved citations were exported into Microsoft Excel for initial screening. Duplicates were removed, and the titles and abstracts were screened by two independent reviewers for relevance. The full texts of potentially eligible studies were obtained and reviewed against the inclusion and exclusion criteria. Discrepancies in selection decisions were resolved through discussion and, where necessary, consultation with a third reviewer. The study selection process was documented and presented using a PRISMA flow presented in Figure 1, including the number of studies identified, screened, excluded, and finally included.

### Data Extraction

A standardised data extraction form was developed to ensure consistency in data collection. The following variables were extracted from each study: authors, publication year, institutional affiliation, study design, sampling method, participant characteristics, prevalence of e-cigarette use, awareness and knowledge levels, and reported influencing factors. Two reviewers independently performed data extraction, and a third reviewer validated the accuracy and completeness of the data.

### Quality Assessment

The methodological quality of the included studies was assessed using the Joanna Briggs Institute Critical Appraisal Checklist for Analytical Cross-Sectional Studies (27). The checklist evaluated nine domains, including inclusion criteria clarity, subject and setting descriptions, valid and reliable measurement of exposure and outcomes, confounder identification, and use of appropriate statistical analysis. Each item was rated “Yes,” “No,” “Unclear,” or “Not Applicable.” The quality appraisal results were used to inform the interpretation of the findings but not as exclusion criteria for study inclusion.

### Data Synthesis and Analysis

Given the heterogeneity of the study populations, operational definitions of e-cigarette use and outcome reporting, a meta-analysis was not feasible. Instead, a narrative synthesis approach was employed. Findings were grouped according to the following thematic domains: (i) prevalence of e-cigarette use, (ii) awareness and knowledge levels, (iii) attitudes and behavioural trends, and (iv) associated influencing factors. Trends were identified and discussed in relation to institution sampling, study method, participant’s gender, and study findings. Particular attention was paid to emerging patterns in influencing factors such as peer pressure, media exposure, and perceived harm of using e-cigarettes.

### Ethical Considerations

This study analysed data extracted from publicly available literature. No human participants were directly involved; hence, ethical approval was not required.

## Results

**Figure 2:**
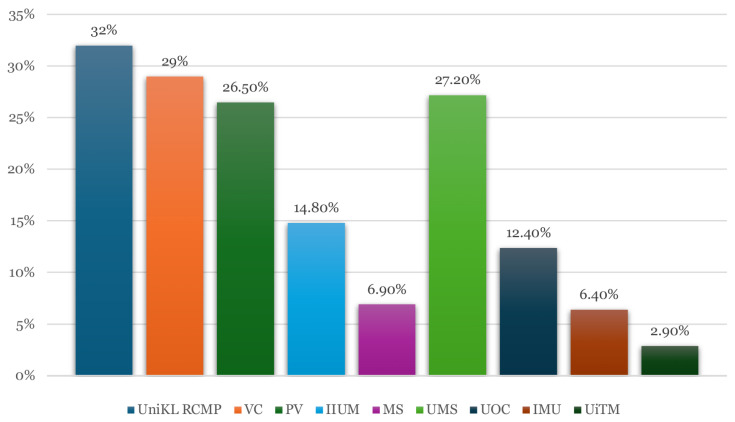
Percentage of vaping prevalence in Malaysian institutions UniKL RCMP = Universiti Kuala Lumpur Royal College of Medicine Perak; VC = vocational college; PV = private college; IIUM = International Islamic University Malaysia; MS = medical school; UMS = Universiti Malaysia Sabah; UOC = University of Cyberjaya; IMU = International Medical University; UiTM = Universiti Teknologi MARA

The initial database search yielded 476 records. After removing 112 duplicates, 364 titles and abstracts were screened for relevance. Of these, 41 articles underwent full-text review. Ultimately, nine studies met the inclusion criteria and were included in this systematic review. All selected studies were cross-sectional in design and were conducted between 2022 and 2024, focusing on e-cigarette use among Malaysian student populations. Figure 1 shows the PRISMA flow diagram illustrating the selection process, and Table 1 summarises the characteristics of the included studies. Meanwhile, Figure 2 illustrates the prevalence rate of vaping across Malaysian educational institutions.

The nine studies included in this review involved a cumulative sample of 2,828 students across public and private tertiary education institutions in Malaysia. Sample sizes ranged from 145 to 614 participants. The participants’ ages ranged from 17 to 40 years, with most studies focusing on the 18 to 25 age group. Table 1 presents the summary of the systematic review study. Participants were enrolled in various academic programmes, including diploma, undergraduate, and postgraduate levels across faculties, such as medicine, nursing, dentistry, and vocational studies. This systematic review synthesised recent evidence on the prevalence, knowledge, attitudes, and factors associated with e-cigarette use among Malaysian students. The reviewed studies demonstrated a wide variation in e-cigarette use prevalence, ranging from 2.9% to 32.0%. Notably, higher prevalence rates were observed among students from non-healthcare backgrounds, whereas medical, nursing, and dental students consistently reported lower usage rates; this disparity may reflect differences in health literacy, educational exposure, and risk perception across academic disciplines.

Table 2 provides a comparative summary of the sample characteristics, institutional contexts, prevalence rates, and key findings from the nine studies included in this review. Notably, the prevalence of e-cigarette use varied widely across institutions, ranging from 2.9% among dental students at Universiti Teknologi MARA to 32.0% among students at Universiti Kuala Lumpur Royal College of Medicine Perak (28). The table highlights a consistent trend: students enrolled in healthcare-related programmes (e.g., medicine, nursing, and dentistry) reported lower usage rates and higher knowledge levels than those in non-healthcare or vocational programmes. For instance, nursing students at UiTM demonstrated not only lower prevalence (14.8%) but also high levels of awareness and preventive practices (30), whereas students from vocational colleges and private institutions without a healthcare focus recorded significantly higher rates, often exceeding 25% (20, 28, 29).

These findings indicate that educational background, particularly in health-related disciplines, may offer a protective effect against e-cigarette use, likely owing to a better understanding of the associated risks and greater exposure to preventive education. Peer pressure, media influence, and perceived stress relief emerged as recurring drivers among institutions with higher usage rates, reinforcing the need for tailored prevention strategies. The stratification of results by institution type and academic discipline provides further insight into the socio-academic dimensions of vaping behaviour among Malaysian students.

### Prevalence of E-Cigarette Use

The prevalence of e-cigarette use among Malaysian students varied considerably across the included studies, ranging from 2.9% to 32%, depending on the institution type, academic programme, and demographic characteristics. Figure 1 summarises the prevalence of vaping in Malaysian institutions, with the highest rate of 32% reported among students at Universiti Kuala Lumpur Royal College of Medicine Perak (28). Similarly, a prevalence of 29.0% was observed among diploma students at a vocational college, strongly associated with social addiction and peer influence (29). In a private college in Sabah, the prevalence of vaping was reported at 26.5% (20). Mokhtar et al. (30) noted a lower prevalence of 14.8% among nursing students, who also exhibited high knowledge levels and proactive prevention behaviours. The lowest prevalence rates were reported in medical and health-related faculties, including 6.9% among medical students (31) and 2.9% among medical and dental students at Universiti Teknologi MARA (35). A prevalence of 27.2% was found among students at Universiti Malaysia Sabah (32), whereas a lower rate of 6.4% was found among students at International Medical University (33). The University of Cyberjaya reported a prevalence of 12.4% (34). These findings suggest that formal education in healthcare-related programmes may offer a protective effect, likely owing to increased awareness of the health risks of vaping. Compared internationally, the prevalence of vaping among students in Malaysia appears moderate, aligning with reports from the United States and Europe, where adolescent vaping rates range between 10% and 27% (36).

### Worldwide Use of E-Cigarettes

The National College Health Assessment III found that 22.1% of undergraduates used e-cigarettes in the past 30 days (37), consistent with the Monitoring the Future study reporting 26% current use among 19- to 22-year-olds (38). Canadian data from the 2019 Tobacco and Nicotine Survey indicated that 26% of young adults aged 20 to 24 years had vaped recently (39). Poland’s large-scale student survey reported 23.4% current users (40), France 21.3% (41), Spain 9.7% (42), and the UK 11% among 18- to 24-year-olds (43). Experimentation peaks under the age of 25, indicating that university students are a key demographic (44). South Korea and China reported rates of 15.2% (45) and 10.3% (46), respectively, with Japan showing lower but increasing rates due to heated tobacco products (47). Malaysia reported 14.7% current users and over 35% lifetime use in some samples (48), Indonesia 10% to 20% among urban university students (49), and the Philippines 13.6% (50). Saudi Arabia (21.3%) (51), Jordan (17.6%) (52), and Lebanon (22.1%) (53) also reported a notable prevalence. South Africa emerged at 13.1% (54, 55) and Nigeria at 9.4% (56). Oceania presents relatively high levels, with Australia at 14.5% (57) and New Zealand exceeding 25% in some urban institutions (58, 59).

### Awareness and Knowledge Levels

Awareness of e-cigarettes was generally high across studies. For instance, 89% of participants in a private college in Sabah were aware of e-cigarettes, whereas 83.8% of the nursing students demonstrated good knowledge. In contrast, 62.1% of medical and dental students exhibited poorer knowledge, although another study reported that 94.4% of medical students had good knowledge, possibly reflecting curricular differences and health promotion efforts. Similar trends exist globally. US undergraduates show over 92% awareness (37), Canadian students 94% (39), and European students over 95% (60). In the UK, awareness was 96%; however, only 59% correctly identified the presence of nicotine (61). South Korea and China reported high awareness but gaps in detailed knowledge (62, 63). Malaysian students showed 94.3% awareness but only 41.2% had accurate knowledge of health effects (64). Studies conducted in Jordan (65), Saudi Arabia (66), Lebanon (67), South Africa (68), Nigeria (56), Australia (69), and New Zealand (57) confirmed high awareness but persistent misconceptions.

### Knowledge Levels and Misconceptions

Despite high awareness, knowledge of e-cigarette contents, health risks, and regulations remains inconsistent. For instance, a study conducted in the United States found that only 60% knew that e-cigarettes contain nicotine, and 28% believed they were harmless (70). Similar gaps exist in Canada (63), France (43), Germany (71), South Korea (45), China (67), Southeast Asia (68), the Philippines (50), Saudi Arabia (51), Jordan (52), South Africa (53), Nigeria (54), Australia (55), and New Zealand (56). These misconceptions may contribute to higher prevalence and risky behaviours, emphasising the need for targeted education.

### Attitudes and Perceived Harm

Student attitudes toward vaping are influenced by perceived harm. Sinnathamby et al. (32) reported that 53.7% believed that e-cigarettes were less harmful than traditional cigarettes. Students with higher knowledge, especially in health-related fields, tended to perceive e-cigarettes as more harmful and had lower usage rates (31–35). Ali et al. (33) found that 38.9% used vaping as a cessation tool, reflecting harm-reduction beliefs. These perceptions align with the international literature indicating that perceived reduced harm drives vaping uptake among the youth (72). However, the effectiveness of e-cigarettes for cessation remains controversial, particularly owing to dual use patterns among the youth (74).

### Factors Influencing E-Cigarette Use

Peer pressure has consistently emerged as a dominant influence on the initiation and continuation of vaping, as cited in at least five studies (20, 29–33). Social trends and lifestyle alignment further affected use, particularly in general student populations (28). Ronald Eden et al. (20) and Mahamad Sobri et al. (35) noted exposure to media promoting vaping. Abd Hadi et al. (31) identified stress relief as a motivator, especially among medical students. Personality traits such as low extraversion increased vaping risk, whereas higher parental income served as a protective factor (23, 34). These findings are consistent with behavioural research emphasising social influence in collectivist cultures such as Malaysia (74). Peers, social media, and online forums remain the dominant information sources, with minimal input from academic or healthcare sources—a pattern also seen in US studies where over 70% learned about vaping socially (75).

### Cessation Practices and Preventive Attitudes

Few studies have reported on cessation-related practices. Mokhtar et al. (30) found that 98.5% of nursing students practiced good prevention and cessation behaviours, possibly owing to curricular emphasis. However, institutional policies or interventions were not documented. Internationally, cessation attempts are common but largely unsupported: in the US, 61% of e-cigarette users tried to quit but only 12% succeeded long-term (76); in the UK, 25% tried quitting but only 15% used formal cessation aids (77). In Malaysia, 45% of students attempted to quit and only 14% sought professional help (78). South Korean data similarly report high quit attempts but frequent failure because of lack of support (79). Social contexts often encourage continued use, with vaping viewed as socially acceptable in university settings (80, 81).

### Preventive Attitudes Toward E-Cigarette Use

Attitudes vary globally. Miech et al. (71) found that 68% of US college students viewed e-cigarettes as harmful, though misconceptions persist. In the UK, 70% favoured stricter regulation (82). South Korean students showed less concern, with only 23% worried about vaping dangers (45, 83). Alsanea et al. (51) noted that several Saudi students viewed vaping as effective cessation, downplaying risks. New Zealand students supported bans on flavoured e-cigarettes (84). Australian campaigns increased negative attitudes, with 79% opposing campus vaping (85). However, students generally receive minimal education on vaping harms (57). In Canada, only 17% of universities had vaping prevention programmes (86); this underscores the need for comprehensive regulation and education to curb vaping among the youth (87).

### Evolving Perceptions of Vaping in Malaysia

The findings of this study significantly contribute to the evolving landscape of vaping-related research in Malaysia by offering updated insights into the current levels of knowledge, attitudes, and awareness among specific population groups. When compared with earlier studies conducted over the past decade, several key divergences and continuities emerge that warrant critical attention. First, the present study shows a moderate to high level of awareness about the health risks associated with vaping, a notable shift from early studies conducted between 2014 and 2016, which reported relatively low public knowledge on the constituents of e-cigarette aerosols and their potential long-term health effects (88). This upward trend in awareness may be attributed to the increasing media coverage, public health campaigns, and recent policy debates surrounding Malaysia’s vaping regulation. However, despite this enhanced awareness, misconceptions persist, particularly around the belief that vaping is a safe alternative to traditional tobacco smoking. This mirrors findings from mid-decade research (89), indicating that while factual knowledge has improved, risk perception has not evolved at the same pace. This misalignment between knowledge and perception poses challenges for public health messaging and indicates the need for more targeted education interventions.

In terms of attitudes, our study found a somewhat more neutral or ambivalent stance among young adults toward the use of vape products, in contrast to earlier findings that indicated stronger pro-vaping sentiments (90). The gradual tightening of vape regulations in Malaysia, combined with a broader shift in societal norms regarding vaping, could influence the change. Notably, public discourse linking vaping to youth nicotine addiction has increased in recent years, which may be reshaping attitudes, particularly among university-aged individuals. Furthermore, a comparison with the findings from a national-level survey in 2020 (91) highlights a concerning disparity in awareness levels between urban and rural populations, a trend that remains consistent in our current data. While urban respondents show relatively higher knowledge and awareness scores, rural populations continue to lag, emphasising the persistent digital and informational divide that influences health literacy.

The evolution of vaping behaviours, particularly among adolescents and young adults, also mirrors global trends. Our findings echo international literature (92), which suggests that vaping is increasingly normalised among youth, despite growing awareness of its risks. In the Malaysian context, this normalisation appears to be facilitated by the easy availability of vape products and the influence of social media marketing, a factor highlighted in several recent local studies (93). Overall, this indicates that while knowledge and awareness have gradually improved over the past decade, translating that knowledge into healthier attitudes and behaviours remains challenging. The complex relationship between information, perception, and behaviour in the context of vaping underscores the requirement for multifaceted public health strategies that go beyond awareness campaigns to address the socio-cultural and economic drivers of vape use.

### Digital Marketing and Online Availability of E-Cigarettes

Digital marketing has emerged as a powerful driver of e-cigarette use in Malaysia, with online platforms shaping perceptions, awareness, and initiation among young people. Analyses of websites of Malaysian e-cigarette retailers revealed the frequent use of promotional claims such as “reduced harm,” “smoke-free lifestyle,” and “socially acceptable alternative,” alongside attractive flavour descriptors and discount offers (94). Notably, several sites failed to provide clear nicotine content information or adequate age-verification mechanisms, raising concerns about access for the youth. Social media platforms further amplify these marketing tactics through influencer endorsements, peer sharing, and targeted advertisements, which normalise vaping and portray it as fashionable or less harmful than conventional cigarettes (7). Such digital promotion strategies undermine public health messaging and contribute to the growing prevalence of vaping among adolescents and young adults.

Beyond marketing, online availability has also facilitated widespread access to e-cigarettes despite regulatory restrictions. Although the Control of Smoking Products for Public Health Act 2024 prohibits online sales and mandates strict registration and labelling requirements, enforcement gaps persist (95). E-cigarettes remain accessible through e-commerce platforms, social media marketplaces, and cross-border shipments, creating loopholes that limit the effectiveness of current policies. This persistent availability underscores the requirement for comprehensive digital surveillance, greater collaboration between regulators and online platforms, and stronger enforcement mechanisms to curb illegal sales.

Complementary public health interventions, such as counter-marketing campaigns and youth-focused education delivered via the same digital platforms, are essential to reduce the appeal of vaping and challenge industry-driven narratives. Addressing these digital dimensions is crucial to the success of Malaysia’s e-cigarette control strategies.

### Limitations

All included studies were cross-sectional, limiting the ability to infer causality. Self-reported measures may introduce recall and social desirability biases. In addition, most studies focused on specific institutions or regions, which may limit their generalizability to the broader Malaysian student population. Heterogeneity in measurement tools, vaping definitions, and knowledge scales also complicates direct comparison across studies. Furthermore, gender-specific analysis and longitudinal data are lacking, and future research should address these gaps to understand usage trajectories and intervention outcomes better.

### Recommendations for Future Research

There is a need for longitudinal studies to track changes in vaping behaviour over time, particularly in response to public health campaigns and regulatory changes. Moreover, mixed-method approaches could help uncover the deeper socio-cultural drivers of e-cigarette use. Future research should explore the intersection between vaping and mental health, as well as the impact of emerging products, such as disposable vapes and synthetic nicotine devices.

## Conclusion

This systematic review achieves its objective by offering a comprehensive synthesis of the prevalence, knowledge, attitudes, and determinants of e-cigarette use among Malaysian students. The evidence shows a troubling usage level, shaped by knowledge gaps, misperceptions of harm, peer and media influences, and stress-related coping behaviours. The disparity between students in health-related and non-health-related fields further underscores the need for targeted interventions. These findings emphasise the urgency of implementing multifaceted public health strategies, encompassing education, policy, media regulation, and cessation support, to curb e-cigarette use. This review serves as a critical evidence base for shaping effective prevention efforts and guiding national health initiatives aimed at safeguarding youth well-being.

## Figures and Tables

**Table 1:** Summary of the studies

Author	Institution sampling	Participants	Result
Ronald Eden et al. (20)	Private college, Sabah	74 male and 171 female students aged 18 to 30 years enrolled in degree, diploma and foundation programmes	Awareness of e-cigarettes is high (89%) 26.5% participants are vaping Internet and peers’ influence
Htwe et al. (28)	Universiti Kuala Lumpur Royal College of Medicine Perak	101 male and 143 female students aged 18 to 30 years enrolled in degree, diploma and foundation programmes	Most volume of e-cigarettes consumed is 10.1 mL to 15.0 mL 32% participants vaping Social trend
Mohd Shoaib et al. (29)	Vocational college, Malaysia	340 male and 274 female students aged 18 to 25 years enrolled in diploma programme	29.0% participants vaping 88.6% agree that e-cigarettes emerge as a new form of society addiction Peers influence
Mokhtar et al. (30)	International Islamic University Malaysia	86 male and 184 female students aged 20 to 24 years enrolled in the diploma programme	83.8% have good knowledge of vaping/smoking 14.8% were active/ex vaping/smoking 98.5% have good practice towards vaping prevention and cessation
Abd Hadi et al. (31)	Medical schools, Malaysia	141 male and 236 female medical students aged 17 to 25 years	6.9% participants vaping 94.4% have good knowledge of e-cigarettes Peer pressure and stress relief
Sinnathamby et al. (32)	Universiti Malaysia Sabah	66 male and 38 female students aged 18 to 40 years in various faculties	27.2% participants are vaping 53.7% believed that e-cigarettes are less harmful Peer pressure and cigarette smoking
Ali et al. (33)	University of Cyberjaya, Selangor	51 male and 94 female students aged 18 to 25 years in various faculties	12.4% participants are vaping 38.9% use vape for smoking cessation Peer pressure
Yew Shen et al. (34)	International Medical University, Kuala Lumpur	117 male and 303 female undergraduate and postgraduate students	6.4% participants are vaping High parents’ annual income prevents vaping Lower extraversion in personality traits
Mahamad Sobri et al. (35)	Universiti Teknologi MARA, Selangor	144 medical and 165 dental students aged 18 to 25 years	2.9% vaping 62.1% have poor knowledge of vaping Media exposure

The study method used in all studies was a cross-sectional, questionnaire-based design

**Table 2:** Comparative summary of e-cigarette use

Author	Sample size	Institution type	Faculty/programme	Age range (years)	Notable findings
Ronald Eden et al. (20)	204	Private	Non-healthcare	17 to 25	Low knowledge; social trends drive use
Hwe et al. (28)	318	Private	Mixed	18 to 35	Highest prevalence; low health literacy
Mohd Shoaib et al. (29)	243	Public	Vocational/diploma	18 to 25	Peer/social influence dominant
Mokhtar et al. (30)	298	Public	Nursing	18 to 26	High awareness; proactive prevention
Abd Hadi et al. (31)	216	Public	Medical	20 to 30	Used for stress relief; good knowledge
Sinnathamby et al. (32)	614	Public	Mixed programmes	18 to 27	Misconceptions common; peer influence noted
Ali et al. (33)	314	Private	Medical	18 to 25	Good knowledge; cessation attitude high
Yew Shen et al. (34)	345	Private	Mixed (non-healthcare)	19 to 26	Moderate prevalence; media exposure noted
Mahamad Sobri et al. (35)	276	Public	Medical/dental	19 to 24	Lowest prevalence; high awareness
